# Determination of ADH in textiles using the HPLC-MS/MS method and the study of its adsorption behaviour towards formaldehyde†

**DOI:** 10.1039/c7ra13155k

**Published:** 2018-01-15

**Authors:** Jinxiong Tao, Ziwei Lin, Haixuan Zhang, Zhuoming Wu, Haihui Cao

**Affiliations:** Shenzhen Academy of Metrology and Quality Inspection Shenzhen 518000 P. R. China taojinxiong@yeah.net; Embry (China) Garments Co. Ltd Shenzhen Guangdong 518000 P. R. China

## Abstract

In the textile industry, formaldehyde-based resins are used as finishers to make the fabrics crease-resistant, which are the main source of formaldehyde in textiles. In our practical study, there are cases that prove that textile products containing adipic dihydrazide (ADH) will continuously adsorb formaldehyde from the surrounding environment during storage. In this study, a high performance liquid chromatography-tandem mass spectrometry method was established for the precise determination of ADH in textiles. The method was optimized in terms of instrument conditions, extraction temperature, extraction time, and extraction mode. Under optimum test conditions, ADH was determined precisely with the linearity range of 0.05–2 mg L^−1^ and correlation coefficient (*R*^2^) of 0.9993. Recovery rate and repeatability were tested; the data showed that the recovery rate of ADH in textiles was in the range of 85–100%, and the RSD (relative standard deviation) was less than 10%. The ADH-positive textile samples were placed in designed environments for some time to adsorb formaldehyde. The adsorbed amounts of formaldehyde in the textile samples first increase and then decrease with time. The maximum amount of formaldehyde a sample can adsorb increases with an increase in its ADH content and will stop increasing once its ADH content exceeds 1700 mg kg^−1^. The placement environment has a little effect on the maximum adsorption capacity of the samples towards formaldehyde, but can significantly affect the adsorption rate and equilibrium adsorption capacity.

## Abstract 2

Study on the adsorption behaviour of textiles containing ADH towards formaldehyde under different placement environments.

## Graphical abstract

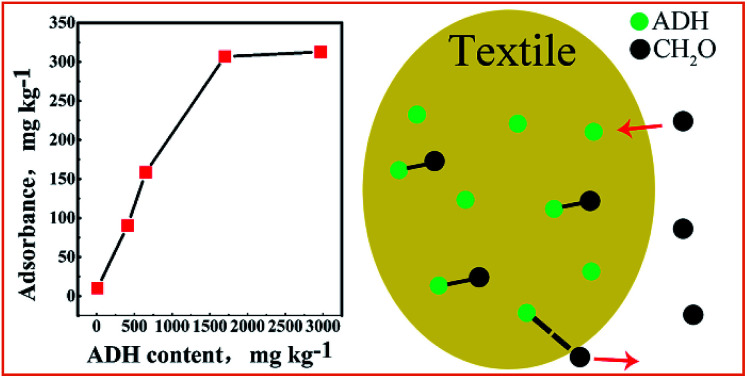

## 1 Introduction

Adipic dihydrazide (ADH) is a white powdery compound with a high melting point, which is produced by the reactions of adipic acid or its derivatives with hydrazine. It is mainly used as an epoxy powder coating curing agent, coating agent, metal deactivator, polymer additive, and water treatment agent. As a bi-functional cross-linking reagent,^1–4^ ADH can react with aldehydes to form relatively stable hydrazone links.

In the textile industry, ADH is commonly used as an anti-yellow auxiliary agent in the process of high-temperature stereotyping of textiles. Due to its active chemical properties, ADH can react with formaldehyde by acylation and often serves as a formaldehyde adsorbent. This characteristic provides the ADH-containing textiles the ability to continuously adsorb formaldehyde even in an environment with a very low formaldehyde content. There are cases in our practical study that confirm the deduction. The textile products with added ADH do not show the existence of formaldehyde in initial factory inspection; however, after a period of storage, transportation, and display, the formaldehyde content of these products can even exceed the restriction requirement of GB18401-2011 (ref. 5) (a mandatory national standard of China) and Oeko-Tex Standard 100-2017.^6^

Formaldehyde is an organic compound. It was formerly used as a disinfectant and preservative of biological specimens. The textile industry uses formaldehyde-based resins as finishers to make the fabrics crease-resistant.^7^ The textiles containing formaldehyde will gradually release free formaldehyde during use and wear. Formaldehyde can cause respiratory inflammation and inflammation of the skin through bodily contact and can also irritate the eyes.^8–10^ The maximum daily reference dose (RfD) for formaldehyde set by the United States Environmental Protection Agency is 0.2 mg kg^−1^. Due to its widespread use, toxicity, and volatility, exposure to formaldehyde significantly affects human health. In 2011, the US National Toxicology Program described formaldehyde as a human carcinogen.

To date, many efforts have been devoted to the study of detection and emission of formaldehyde from our living environments.^11–14^ However, no method has been developed for the determination of ADH in textiles, and its absorbability towards formaldehyde has not been studied to date. Therefore, it is necessary to develop a method to detect ADH in textiles. In analytical chemistry, UV spectrophotometry,^15^ fluorescence spectrophotometry,^16^ GC/MS,^17^ HPLC/MS,^18^*etc.* are the frequently used tools for quantitative analysis. In this study, a high performance liquid chromatography tandem mass spectrometry method was established to detect ADH. The chromatographic conditions, extraction mode, extraction solvent, and extraction temperature and time were optimized for the precise detection of ADH content with high sensitivity. Moreover, we explored the adsorption behaviour of the textiles containing ADH towards formaldehyde to find out the relationship between the ADH content in the textiles and formaldehyde adsorption.

## 2 Materials and methods

### 2.1 Chemicals and reagents

Standards of adipic dihydrazide (ADH, 96%, *M*_w_ = 175.2) and formaldehyde (10.5 mg mL^−1^, *M*_w_ = 30.03) were purchased from Toronto Research Chemicals Inc. and National Institute of Metrology, Beijing, China, respectively. HPLC grade methanol and acetonitrile were supplied by Merck Holding Ltd, Shanghai, China. HPLC grade formic acid and ammonium acetate were purchased from Aladdin Reagent, Shanghai, China. Ultrapure water was prepared by a Milli-Q with an electrical resistivity of 18.2 MΩ. Other reagents were obtained from commercial sources and used as received. ADH-positive textile samples were provided by the clients. The samples were prepared by immersing the textile cloth in solutions with different ADH contents and then drying. All the samples were nylon/spandex blend fabrics with nylon content ranges of 80–90% and differed in the weaving structure.

### 2.2 Instruments

An Agilent 1290-G6490 high performance liquid chromatography-tandem mass spectrometer (HPLC-MS/MS) was used for the measurement of ADH. The Agilent Cary 60 UV-vis spectrophotometer was used for detection of formaldehyde. Samples were weighed by a Mettler Toledo XA205 analytical balance. The ultrasonic cleaner made by KUDOS was used to extract ADH from textiles.

### 2.3 HPLC-MS/MS conditions

ADH contents of all the samples were analyzed using the HPLC-MS/MS system equipped with a 3.0 mm × 100 mm Poroshell Hilic column with a bead size of 2.7 μm. The column temperature and flow rate were 40 °C and 0.3 mL min^−1^, respectively. The mobile phase consisted of 80% water containing 0.1% formic acid (eluent A) and 20% acetonitrile (eluent B) under isocratic elution. For each sample, a volume of 1 μL was loaded onto the column. For the mass spectrometry detector, data were obtained using the multiple reaction monitoring positive mode with the precursor ion of 175.2 and product ions of 143.1, 115.0, and 111.1.

### 2.4 Detection and determination of ADH in textiles

The textile specimen was cut into pieces (approximately 5 mm × 5 mm). About 1 g of the cut textile was prepared and placed in a glass container. Then, 10 mL of water was pipetted into the container that was then placed in an ultrasonic bath at 80 °C for 90 min. After this, the extract was filtered into an HPLC vial and analyzed by HPLC-MS/MS. Each sample was tested twice, and the results were averaged.

### 2.5 Determinations of the adsorbed formaldehyde content in the textiles containing ADH

Textile products with added ADH usually adsorb a certain amount of formaldehyde from the surroundings after being stored for a period of time. Herein, two kinds of testing environment were set to investigate the adsorption behaviour of the ADH-positive samples towards formaldehyde: (a) laboratory with a constant temperature (20 ± 2 °C) and humidity (65 ± 2%) built according to ISO 139-2005 (ref. 19) and (b) a normal room that was decorated for a few months with the temperature and humidity ranges of 22–28 °C and 40–55%, respectively, during the experimentation period. The samples were stored in the set environments for some time and then prepared and tested according to ISO 14184-1-2011.^20^ The testing procedures were as follows: the samples were cut into small pieces and extracted in water for 60 min at 40 °C. Afterward, the filtered extract solution was transferred into a tube, and acetylacetone reagent was added; then, the solution was quantitated by the Agilent Cary 60 UV-vis spectrophotometer *via* the calibration curve of formaldehyde standard.

## 3 Results and discussion

### 3.1 Optimum test and extraction conditions of ADH

**Fig. 1:**
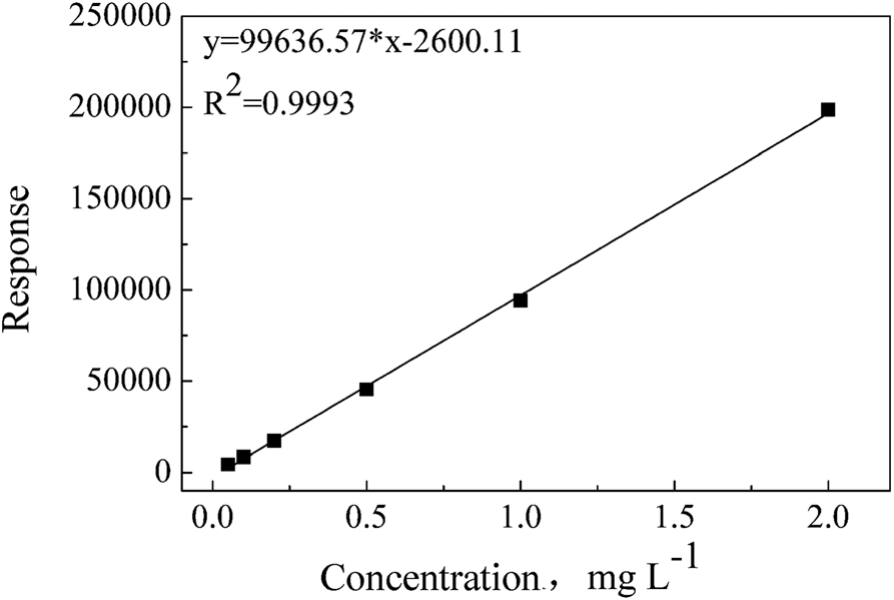
Calibration curve of ADH.

**Fig. 2:**
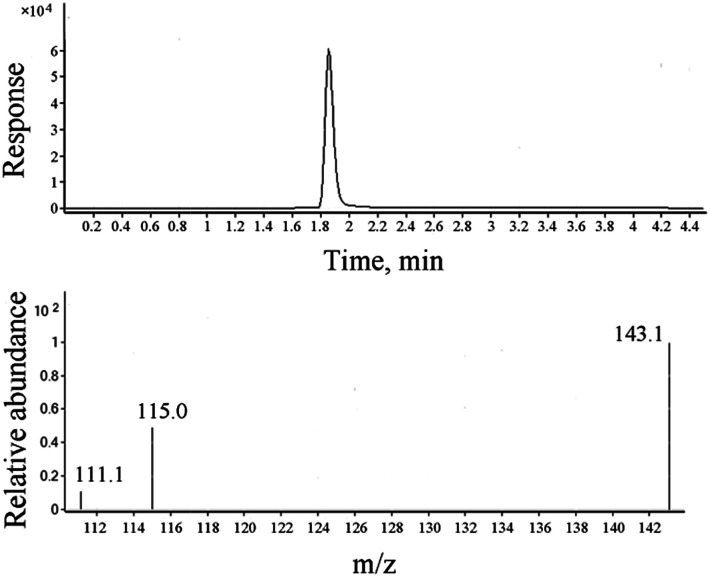
Chromatogram and mass spectrum of ADH.

ADH has better solubility in water than in organic solvents; thus, water has been chosen as an extraction solvent and standard matrix. During the optimization process of chromatographic conditions, it has been found that ADH has no retention in C18 or C8 columns, but can be well retained in a Hilic column (displayed as Fig. S1†). Under the optimum test conditions, ADH was determined precisely with the linearity range of 0.05–2 mg L^−1^ and correlation coefficient (*R*^2^) of 0.9993, as shown in Fig. 1. Repeatability and recovery rate were tested, and the results are listed in Table S1.† Data show that the recovery rate of ADH is in the range of 85–100%, and the RSD (relative standard deviation) is less than 10%. Chromatogram and mass spectrum of ADH are shown in Fig. 2.

Soxhlet extraction, shaking bath extraction, and ultrasonic extraction are the three commonly used traditional methods to extract substances. Since the solvent is water in this study, Soxhlet extraction is not applicable. A comparison between the extraction efficiencies of shaking bath extraction and ultrasonic extraction was made, and the results are shown in Table S2 and Fig. S2.† The data indicate that ultrasonic extraction is more efficient to extract ADH from the textiles.

**Fig. 3:**
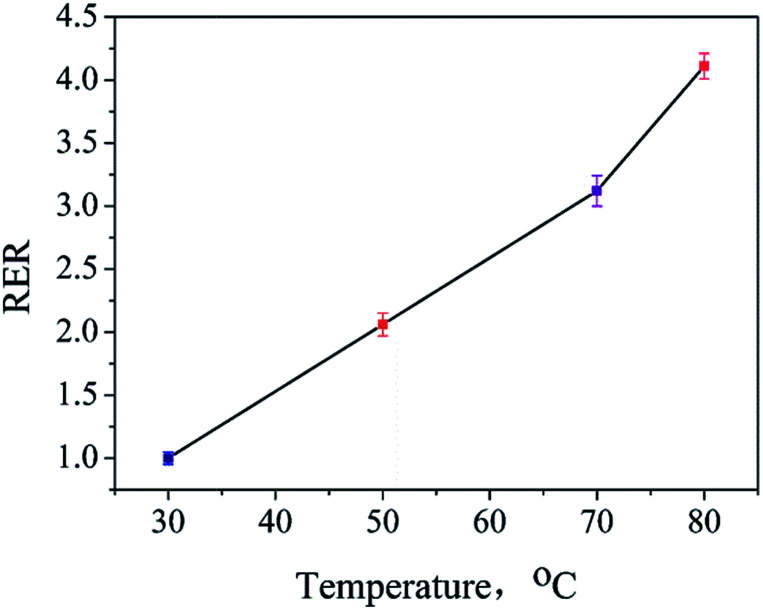
Changes in the RER values over the extraction temperatures.

The influences of temperature and time on the extraction of ADH have been discussed. An ADH-positive sample (S0, shown in Fig. S3(a)†) was prepared and extracted for 1 h at 30 °C, 50 °C, 70 °C, and 80 °C. The extracts were analyzed, and the ADH contents were determined. The ratio between the test result obtained at a certain temperature and the result obtained at 30 °C was defined as the relative extraction rate (RER). As shown in Fig. 3, the RER value increased quickly with an increase in the extraction temperature at the set extraction time. Moreover, 80 °C, which is the highest set point of ultrasonic bath, provides highest RER value and has been chosen to be the best extraction temperature.

**Fig. 4:**
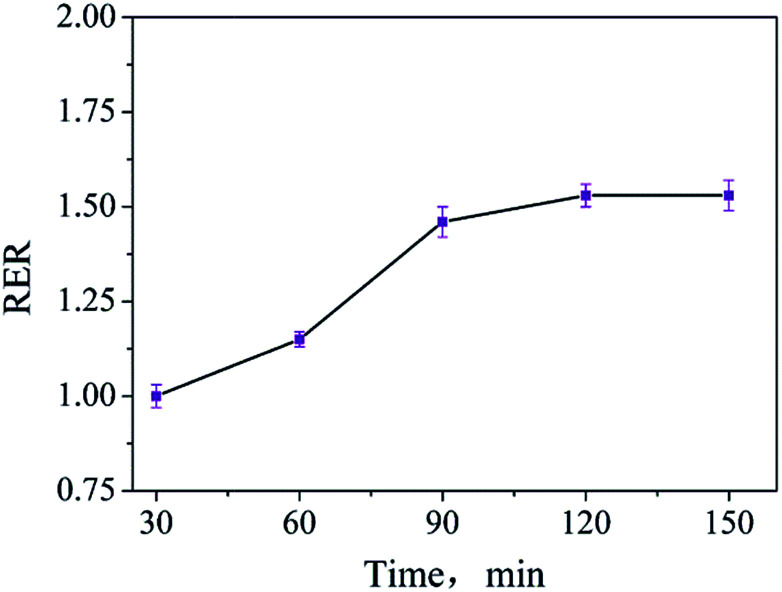
Changes in the RER values over the extraction times.

For optimizing the extraction time, the sample was prepared and extracted at 80 °C for 30 min, 60 min, 90 min, 120 min, and 150 min. Similarly, RERs were calculated from the test results of the samples extracted for different times and those extracted for 30 min. The change in the RER value over the extraction time is shown in Fig. 4. It can be seen that the RER values no longer increase when the extraction time exceeds 120 min; this reveals that the whole amount of ADH is extracted from the tested sample. The difference between the RER values obtained at 90 min and 120 min is small. Considering the extraction effectiveness and efficiency, the extraction time of ADH is best set as 90 min.

### 3.2 Placement environment of the samples

In this study, we have designed two environments for placing the samples: one is a laboratory with a constant temperature and humidity used for the pre-treatment of testing samples and the other is a normal room that has been decorated for a few months. Environment (a) has a constant temperature and humidity and weak air flow, which can be regarded as an enclosed environment. Moreover, its formaldehyde content in air, mainly released from the pre-treating samples, is very low. In contrast, environment (b) exhibits changes in temperature and humidity and has good air circulation, which can be regarded as an open environment. In addition, another consideration was that the room was not decorated long ago; thus, the finishing materials, such as the wall paint and furniture, still had a strong ability to release formaldehyde and thus would give rise to a relatively high formaldehyde content in the air of the local environment. The purpose of setting the two placement environments is to study the influence of temperatures, humidities, and formaldehyde contents of the surroundings on the adsorption behaviour of the textiles containing ADH towards formaldehyde. It is necessary to clarify that in this study, mainly the influence of these factors on the adsorption behaviour within a period of time has been investigated; thus, the real-time formaldehyde content of the surroundings is not monitored.

### 3.3 Relationship between the adsorbed formaldehyde amounts in the textile samples and the ADH contents

Some textile products, especially nylon underwear products, usually have added ADH to avoid yellowing under high-temperature processes. These products will adsorb a certain amount of formaldehyde after being stored for a period of time. The reaction mechanism is that the –NHNH_2_ group of ADH is very active at room temperature and easily reacts with formaldehyde to form a Schiff base by dehydration. The reaction is reversible. If the environment becomes wet, the formed Schiff base can hydrolyze and release formaldehyde. Therefore, adsorption and desorption of formaldehyde by the textiles containing ADH are dynamic equilibrium processes. The reaction mechanism is shown in Fig. S4.†

**Fig. 5:**
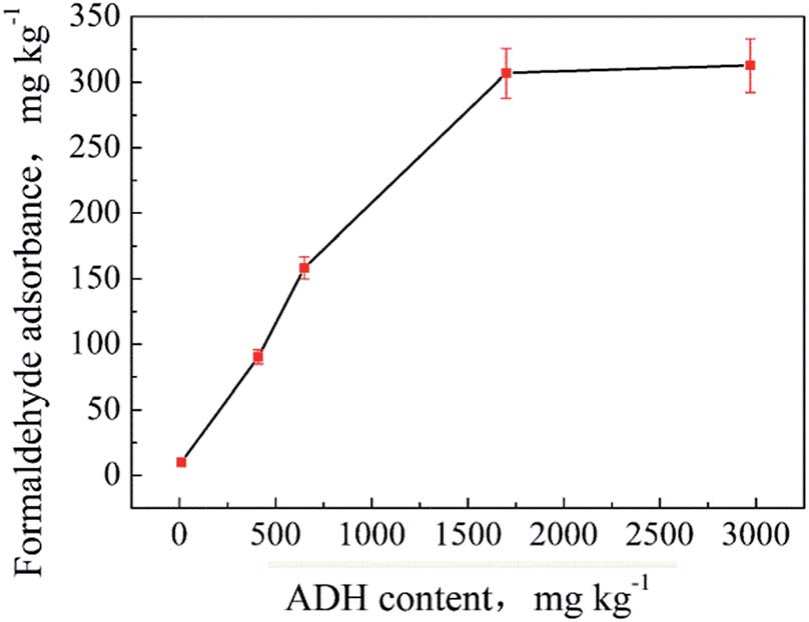
The formaldehyde adsorption capacity of the textile samples along with their ADH contents.

ADH contents of the samples S1–S8 were determined by HPLC-MS/MS. The results are shown in Table 1. The samples were then placed in the environment (a) for a certain time, and their adsorbed formaldehyde contents were determined. The results are listed in Table 1. It can be seen from the table that the samples with an ADH content lower than 10 mg kg^−1^ hardly adsorb formaldehyde or the adsorption quantity is too low to be detected. For the samples with high ADH contents (S3–S8), the adsorbed formaldehyde amounts first show an increasing trend and then a decreasing trend with an increase in the storage time. The maximum amount of formaldehyde adsorbed by a sample is termed as the adsorption capacity. The adsorption capacity *versus* ADH contents is illustrated in Fig. 5. As can be seen, the adsorption capacities of the samples increase with an increase in the ADH contents and show a tendency to slow down as the rate increases. Specifically, the adsorption capacities do not increase once their ADH contents are higher than 1700 mg kg^−1^. These results are not beyond expectation because the active surface area of the textile increases as the ADH content increases; this leads to a greater ability to adsorb formaldehyde from the surroundings. Further, when the ADH content reaches a limit, the adsorption capacity of the textile sample towards formaldehyde will stop increasing due to the saturation of its active surface area. Another phenomenon that cannot be ignored is that the weaving method and structure of the textile fabric have a large impact on its adsorption capacity to formaldehyde; this is well confirmed by the test results of the samples S3 and S5. The ADH content and formaldehyde adsorption capacity of the sample S3 are 540 mg kg^−1^ and 82.3 mg kg^−1^, respectively, whereas the corresponding results of the sample S5 are 410 mg kg^−1^ and 90.2 mg kg^−1^, respectively. Sample S5 has a lower ADH content but higher formaldehyde adsorption capacity than S3. In fact, sample S5 is woven thinner and more loosely than S3 (as shown in Fig. S3(c) and (d)†); this can be the reason for the inconsistent data. Because a loose and thinner structure of the textile increases its surface area and brings a more even distribution of the added ADH on the surface, the active sites are consequently increased and bring a larger adsorption capacity.

**Table 1:** The adsorbed formaldehyde amounts of the testing samples under the conditions of the environment (a)^a^

Sample	Colour	ADH content (mg kg^−1^)	Formaldehyde content (mg kg^−1^)						
			Initial	10 d	30 d	60 d	90 d	120 d	150 d
S1	White^b^	N.D^c^	N.D^d^	N.D	N.D	N.D	N.D	N.D	N.D
S2	White	10 ± 1	N.D	N.D	N.D	N.D	N.D	N.D	N.D
S3	White	540 ± 6	N.D	53.6 ± 4.2	82.3 ± 5.2	63.8 ± 3.7	45.8 ± 1.8	39.7 ± 2.1	38.1 ± 2.8
S4	White	930 ± 7	N.D	65.1 ± 2.6	109.0 ± 6.9	87.9 ± 4.5	63.9 ± 2.8	47.9 ± 2.0	46.0 ± 3.0
S5	Blue	410 ± 3	N.D	56.5 ± 2.7	90.2 ± 7.9	68.6 ± 3.3	50.5 ± 2.3	42.0 ± 2.1	41.1 ± 2.7
S6	Blue	650 ± 9	N.D	78.1 ± 4.9	158.4 ± 8.6	147.8 ± 6.4	109.8 ± 6.8	83.7 ± 4.0	81.0 ± 4.4
S7	White	1700 ± 18	N.D	102.4 ± 8.5	215.4 ± 11.8	306.9 ± 14.9	263.1 ± 20.5	217.0 ± 7.8	192.2 ± 8.8
S8	White	2970 ± 22	N.D	89.5 ± 7.1	233.4 ± 9.6	312.7 ± 16.3	295.9 ± 20.9	267.5 ± 17.4	248.1 ± 12.9

a N.D: not detected.

b Samples with same colour are obtained from the same pieces of cloth and have different addition amounts of ADH.

c Detection limit of ADH is set as 5 mg kg^−1^.

d Detection limit of formaldehyde in textiles is 16 mg kg^−1^ according to ISO 14184.1-2011,^20^ herein, it is set as 10 mg kg^−1^.

### 3.4 Adsorbed formaldehyde amounts in the samples under the conditions of the environment (a)

**Fig. 6:**
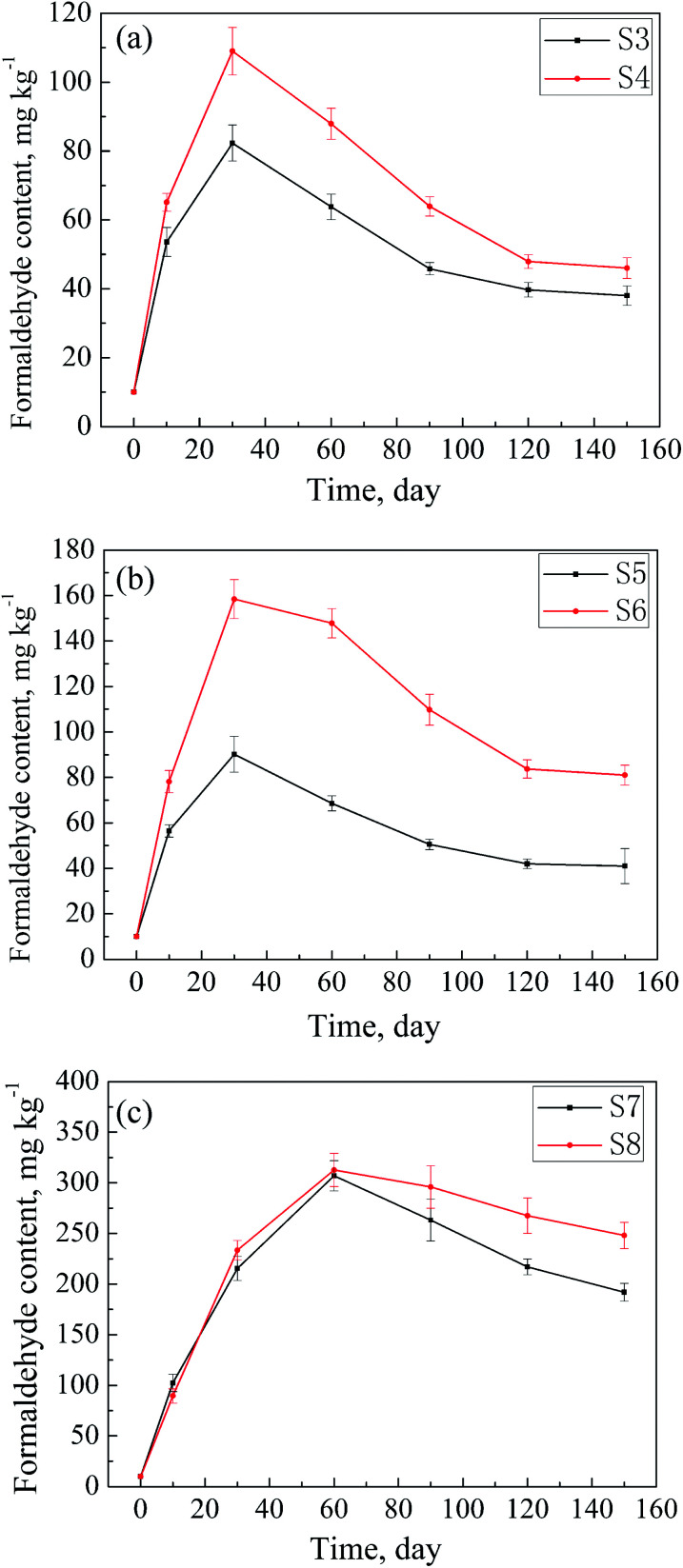
Changes in the adsorbed formaldehyde amounts in the samples S3–S8 over storage time.

The formaldehyde contents of the samples S1–S8 after being placed in the environment (a) for some time were determined and are listed in Table 1. As abovementioned, the adsorbed amounts of formaldehyde in the textile samples increase first and then decrease with time. Changes in the adsorbed formaldehyde amounts over time of the samples S1–S8 are shown in Fig. 6. As can be seen from Fig. 6(a) and (b), the adsorbances of the samples with the ADH contents less than 1000 mg kg^−1^ (S3–S6) reach maximum after 30 days of storage. However, as shown in Fig. 6(c), S7 and S8† adsorb maximum amounts of formaldehyde after being stored for 60 days. The reasonable explanation is that these two samples have high contents of ADH (above 1700 mg kg^−1^), leading to relatively high adsorption rates of formaldehyde in the very beginning. Thus, the adsorbances of the two samples reach a high level in a relatively short time; this results in the acceleration of the desorption rate. The combined contribution of these factors caused an experimental phenomenon such that S7 and S8 needed a longer time to adsorb the maximum amount of formaldehyde. In addition, as illustrated in Fig. 6(a), the amounts of formaldehyde adsorbed by the samples almost don't change after 150 days of storage. Similar conclusions can be drawn from Fig. 6(b). In comparison, as shown in Fig. 6(c), even after storage for 150 days, the adsorbed amounts of formaldehyde of S7 and S8 still show a tendency to decline with time. The data show that the adsorption of textiles with a low ADH content towards formaldehyde can reach adsorption–desorption equilibrium in a relatively short time, whereas it is difficult for the textiles with a high ADH content to reach an adsorption–desorption equilibrium in this set environment.

### 3.5 Adsorbed formaldehyde amounts in the samples under the conditions of the environment (b)

**Fig. 7:**
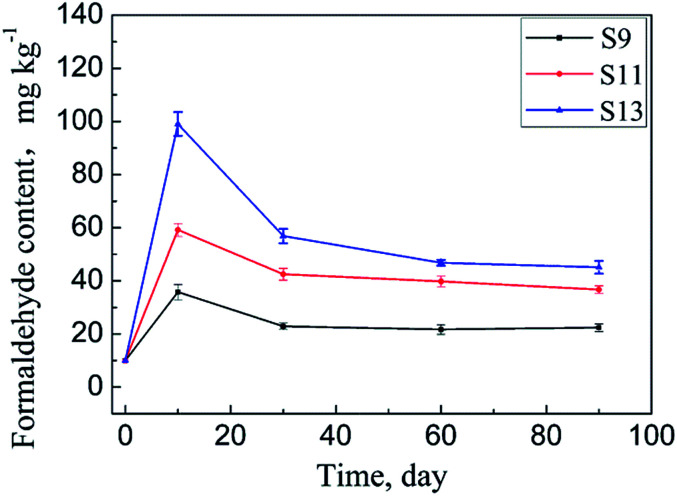
Changes in the adsorbed formaldehyde amounts in the samples S9, S11, and S13 over storage time.

ADH contents of the samples S9–S14 were determined using HPLC-MS/MS. The results are shown in Table 2. The samples were then placed in environment (b) for a certain time, and the adsorbed formaldehyde amounts were determined. The results are listed in Table 2. Changes in the adsorbed formaldehyde amounts of samples S9, S11, and S13 over time are plotted in Fig. 7. Compared with those of the samples S3–S8 in environment (a), the adsorbances of the samples placed in environment (b) also show a similar trend of first increasing and then decreasing, but the difference is that these samples adsorb maximum amounts of formaldehyde after being stored for only 10 days. This result meets the expectation of our design. As stated earlier, environment (b) is a room that has just been decorated, in which the formaldehyde content in air is higher than that of environment (a) due to the formaldehyde release of finishing materials. Further, the temperature of environment (b) is higher than that of environment (a), and the air mobility of environment (b) is also better. The combined effects of these factors lead to a higher formaldehyde adsorption rate for the samples S9–S14 than that for the samples S3–S8.

**Table 2:** The adsorbed formaldehyde amounts of the testing samples under the conditions of the environment (b)

Sample	Colour	ADH content (mg kg^−1^)	Formaldehyde content (mg kg^−1^)				
			Initial	10 d	30 d	60 d	90 d
S9	Green	150 ± 6	N.D	35.8 ± 2.9	22.9 ± 1.3	21.7 ± 1.8	22.4 ± 1.4
S10	Green	372 ± 11	N.D	105.4 ± 7.8	65.6 ± 4.6	54.3 ± 3.7	52.1 ± 2.1
S11	Blue	320 ± 4	N.D	59.2 ± 2.4	42.5 ± 2.2	39.8 ± 1.9	36.7 ± 1.4
S12	Blue	628 ± 9	N.D	146.9 ± 6.6	103.1 ± 7.3	97.2 ± 3.5	95.6 ± 4.2
S13	Purple	576 ± 21	N.D	99.0 ± 4.5	56.9 ± 2.7	46.8 ± 1.1	45.1 ± 2.4
S14	Purple	252 ± 14	N.D	30.0 ± 1.7	19.3 ± 0.8	21.2 ± 1.1	19.6 ± 0.6

As can be seen from Table 2, the sample S12 has an ADH content of 628 mg kg^−1^ and a formaldehyde adsorption capacity of 146.9 mg kg^−1^ under the conditions of environment (b). Moreover, as shown in Table 1, the sample S6 has an ADH content of 650 mg kg^−1^ and a formaldehyde adsorption capacity of 158.4 mg kg^−1^ under the conditions of environment (a). The two samples have not only similar ADH contents but also close formaldehyde adsorption capacities. Considering that they are obtained from the same material, a conclusion can be drawn that the maximum formaldehyde adsorption amounts of the ADH-positive samples are barely influenced by storage environments, but mainly depend on the ADH contents.

As illustrated in Fig. 7, the adsorbed formaldehyde amounts of the samples S9, S11, and S13 nearly stop changing after 90 days of storage, similar to the case of the samples S3–S6. The difference is in the storage time they take to reach the equilibrium. As shown in Tables 1 and 2, sample S6 has an ADH content of 650 mg kg^−1^ and an equilibrium adsorption capacity of 81.0 mg kg^−1^ under the conditions of environment (a), whereas the corresponding data of sample S12 are 628 mg kg^−1^ and 95.6 mg kg^−1^, respectively, under the conditions of environment (b). Compared with S6, S12 has a lower ADH content but larger equilibrium formaldehyde adsorption capacity. Taking into account that the textiles containing ADH have almost the same adsorptive capacity for formaldehyde under the two storage conditions and the humidity of environment (b) is lower than that of environment (a), it can be said that the stability of the Schiff base produced by ADH and formaldehyde is more sensitive to moisture content than to temperature and formaldehyde content in the air of the placement environment. Because, as illustrated before, the reaction is reversible, the existence of too much water vapour in the surroundings will accelerate the reverse reaction.

## 4 Conclusions

In the present study, a high performance liquid chromatography-tandem mass spectrometry method was established for the detection of ADH. The method was optimized in terms of extraction solvent, extraction temperature and time, and LC-MS/MS conditions for the precise determination of ADH in textiles. The adsorption behaviour of textiles containing ADH towards formaldehyde was studied under two designed environments. The formaldehyde adsorption capacities of the samples increase with an increase in the ADH contents in the samples and stop increasing when the ADH contents in the samples exceed 1700 mg kg^−1^. Under constant temperature (20 ± 2 °C) and humidity (65 ± 2%) conditions, the samples with an ADH content below 1000 mg kg^−1^ can adsorb the maximum amount of formaldehyde after being stored for 30 days, whereas those with an ADH content over 1700 mg kg^−1^ need 60 days to adsorb the maximum amount of formaldehyde. For samples placed in a room that has just been decorated, it only takes 10 days to adsorb the maximum amount of formaldehyde. The formaldehyde adsorption capacities of the ADH-positive samples are almost unaffected by the storage environment, but mainly depend on the ADH contents. Moreover, the ambient humidity can significantly influence the equilibrium adsorption amount of formaldehyde in the samples.

## Conflicts of interest

There are no conflicts of interest to declare.

## Supplementary Material

RA-008-C7RA13155K-s001
