# Supplementary material for: Determination of ADH in textiles using the HPLC-MS/MS method and the study of its adsorption behaviour towards formaldehyde
Source: RSC Adv. 2018 Jan 15;8(6):2915–21. doi: 10.1039/c7ra13155k (PMC9077537; doi:10.1039/c7ra13155k)
Supplement: RA-008-C7RA13155K-s001 [file RA-008-C7RA13155K-s001.pdf]

Electronic Supplementary Material (ESI) for RSC Advances.
This journal is © The Royal Society of Chemistry 2017

Supplementary information

Determination of ADH in textiles using HPLC-MS/MS method and the study of its adsorption behaviour towards formaldehyde

Jinxiong Tao,[*,a] Ziwei Lin,[a] Haixuan Zhang,[a] Zhuoming Wu[a] and Haihui Cao[b]

[a] *Shenzhen Academy of Metrology and Quality Inspection, Shenzhen 518000, P. R. China*
[b] *Embry (China) Garments Co. Ltd, Shenzhen, Guangdong, 518000, P. R. China.*
[*] *Corresponding author: E-mail: taojinxiong@yeah.net; Tel: +86-0755-27528470*

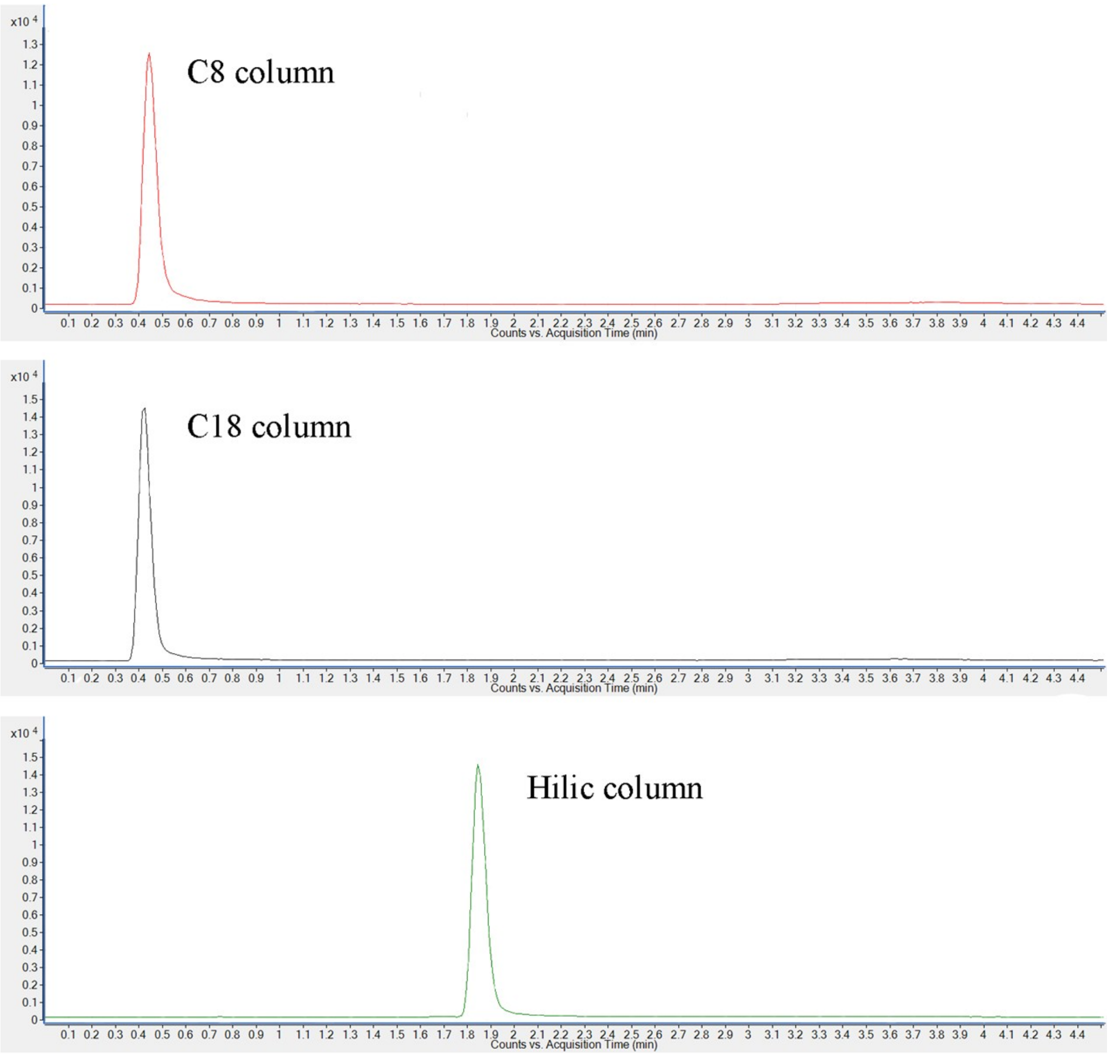

Fig. S1 The separation effect of different columns

Table S1 Determination value of ADH in textiles under different extracting modes

sample	extracting mode	testing results, mg kg^{-1}		
		parallel sample 1	parallel sample 2	average
A	shaking bath extraction	15	15	15
	ultrasonic extraction	16	16	16
B	shaking bath extraction	417	413	415
	ultrasonic extraction	444	441	442
C	shaking bath extraction	650	644	647
	ultrasonic extraction	669	681	675

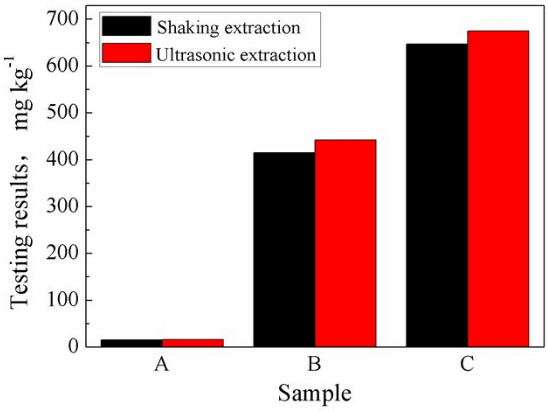

Fig. S2 Comparison of the extraction efficiencies on ADH in textiles under diffenrent extracting modes

Table S2 Recovery rate and RSD values of ADH determination in real samples

Items	Testing results		
Added (mg L^{-1})	0.20	0.50	1.00
Found (mg L^{-1})	0.20	0.43	0.86
	0.19	0.44	0.85
	0.19	0.44	0.86
	0.19	0.43	0.87
	0.19	0.44	0.87
	0.19	0.44	0.87
	0.20	0.44	0.88
	0.20	0.44	0.89
Average value/(mg L^{-1})	0.19	0.44	0.87
Recovery (%)	95	88	87
SD (mg L^{-1})	0.005	0.021	0.045
RSD (%)	2.7	4.8	5.2

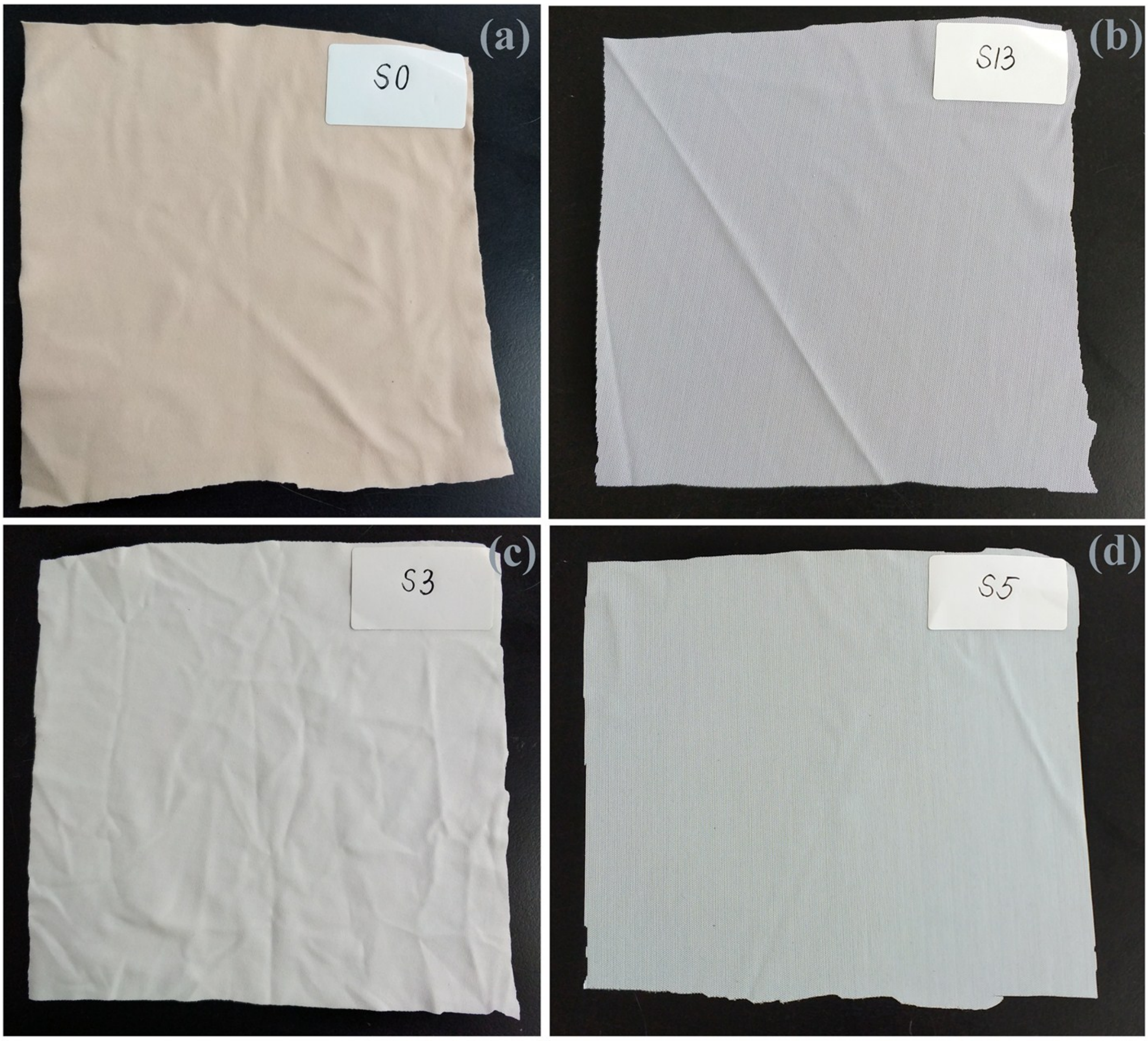

Fig. S3 Photographs of samples S0, S3, S5 and S11

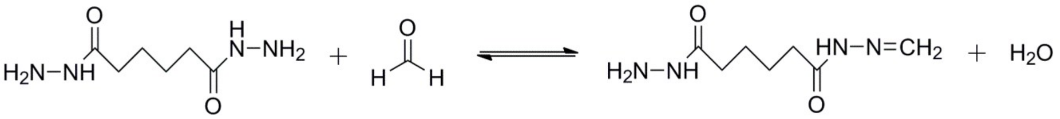

Fig. S4 Reaction mechanism of ADH and formaldehyde in the balance of adsorption and desorption.
